# Progress on the Extraction, Separation, Biological Activity, and Delivery of Natural Plant Pigments

**DOI:** 10.3390/molecules28145364

**Published:** 2023-07-12

**Authors:** Xianwen Lu, Wenjun Li, Qi Wang, Jing Wang, Song Qin

**Affiliations:** 1 Qingdao Academy of Chinese Medical Sciences, Shandong University of Traditional Chinese Medicine, Jinan 250355, China; lxw17863435358@163.com (X.L.); wjli@yic.ac.cn (W.L.); sdlywangqi@163.com (Q.W.); 2 Yantai Institute of Coastal Zone Research, Chinese Academy of Sciences, Yantai 264032, China; elevenwj1111@163.com; 3 School of Pharmacy, Binzhou Medical University, Yantai 264003, China

**Keywords:** plant pigment, chemical classification, extraction method, biological activity, modification method

## Abstract

Natural plant pigments are safe and have low toxicity, with various nutrients and biological activities. However, the extraction, preservation, and application of pigments are limited due to the instability of natural pigments. Therefore, it is necessary to examine the extraction and application processes of natural plant pigments in detail. This review discusses the classification, extraction methods, biological activities, and modification methods that could improve the stability of various pigments from plants, providing a reference for applying natural plant pigments in the industry and the cosmetics, food, and pharmaceutical industries.

## 1. Introduction

The pigment is widely used as an additive in the food, beverage, and cosmetic industries, primarily due to consumers’ pursuit of color. Pigments can be divided into synthetic and natural pigments. William discovered lavender, the first synthetic organic dye, in 1556. Afterward, numerous synthetic pigments were widely used in food, medicine, and cosmetics; however, many toxic substances, such as lead, arsenic, and mercury, have been detected in these synthetic pigments. Since then, the United States has promulgated the Food and Drug Act, which forbids poisonous synthetic pigments as food additives [1]. Natural pigments are safe and non-toxic, and recent research has demonstrated that natural pigments have different biological activities. Natural pigments can be derived from animals, plants, and microorganisms, with plant pigments being the most studied and utilized. Currently, FDA approved synthetic pigments in the United States include red moss red, fast green, bright blue, and sunset yellow; 32 kinds of natural pigments are allowed, such as chlorophyll, beet red, riboflavin, and β-carotene; six kinds of synthetic pigments are allowed in China, and 54 kinds of natural pigments are allowed, indicating that natural pigments continue to be the primary source of pigments. However, natural pigments are less stable than synthetic pigments; therefore, the pigment industry is implementing new technologies to ensure the stability of natural pigments during extraction and application [2].

Pigments extracted from plants are insoluble in water and unstable in light, heat, and at extreme pH levels [3]. Traditional natural pigment extraction techniques, such as solvent extraction, grinding, and pressing, these methods has long time, low yield, need numerous solvents, and some organic solvents are highly toxic [4]. Some green and efficient extraction methods, including (1) supercritical fluid extraction, (2) ultrasound-assisted extraction, (3) enzyme-assisted extraction, (4) microwave-assisted extraction, and (5) ultra high pressure-assisted extraction, have been developed to solve the above problems. Modern studies have confirmed that natural pigments have different biological activities, such as antioxidation, anti-inflammation, anti-cancer, neuro-protection, and cardiovascular protection [5]. Recently, a series of methods have been developed to improve the stability of natural pigments, including the synthesis of lipid-biological macromolecules [6], pigment–protein complexes [7], and pigment-metal nanoparticle synthesis [8].

This review focuses on the chemical structure of natural pigments used in the food and cosmetics industries and the research advancements and biological activity of natural pigment extraction technology.

## 2. Chemical Classification of Natural Pigments

**Figure 1:**
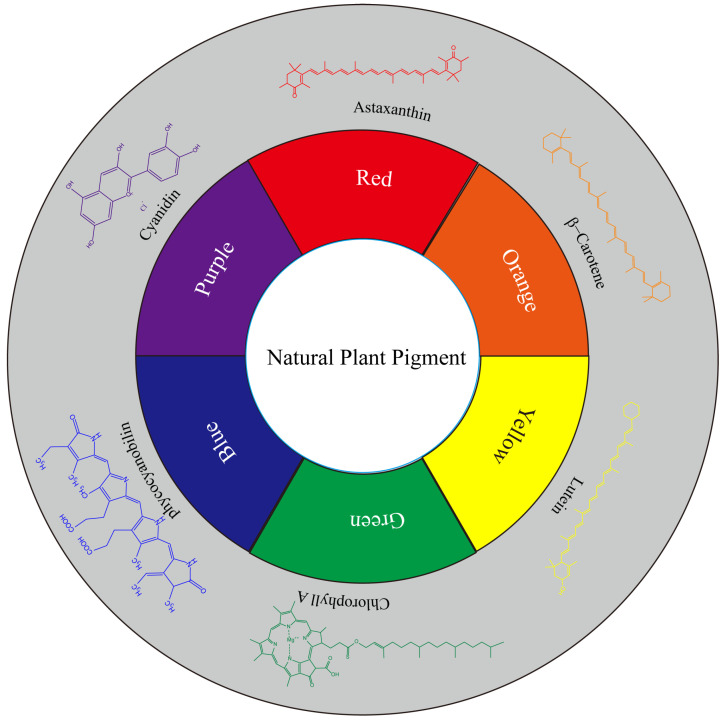
Representative natural plant pigments of different colors: Astaxanthin, β-carrot, Lutein, Chlorophyll A, Phycocyanobilin, and Cyanidin.

According to the color classification, natural pigments can be primarily divided into six categories: red, orange, yellow, green, blue, and purple (Figure 1). According to the chemical structure classification, natural pigments can be primarily divided into five categories: carotenoids, polyphenols, quinones, pyrrole, and pyridines (Table 1).

### 2.1. Carotenoid

Carotenoids, or isoprene derivatives, are conjugated polyene chains that are composed of eight isoprenes [74]. Carotenoids are abundant in animals, higher plants, fungi, and algae. The carotenoid database established by Yabuzaki et al. contains the chemical information of 1117 kinds of natural carrots and 683 kinds of organisms [75]. Carotenoids are primarily yellow, orange, or red. Carotenoids can be divided into two categories based on the different elements they contain: carotene has only carbon and hydrogen atoms, mainly β-carotene and lycopene. One is lutein containing carbon, hydrogen, oxygen, and other factors, primarily astaxanthin, lutein, and zeaxanthin [76]. Carotenoids have various biological activities, such as anti-oxidation, anti-cancer, and immune regulation. Some carotenoids can be used as the premise of vitamin A and have seen great potential for treating vitamin A deficiency [77]. The most important way for humans to intake carotenoids is through fruits and vegetables. The β-carotene mainly exists in green leafy vegetables and yellow or orange fruits or vegetables, such as broccoli, carrots, and mangoes. Lycopene is abundant in tomatoes, watermelons, grapes, and other fruits, but it does not belong to vitamin A and cannot be changed into vitamin A in the body [78].

### 2.2. Polyphenols

Polyphenols are also called plant tannins and can be divided primarily into flavonoids and non-flavonoids. Flavonoids mainly include anthocyanins, isoflavones, and flavonoids: the primary 2-phenylchromone structure as the skeleton. Most flavonoids are fat soluble compounds in water, whereas anthocyanins are water soluble. Non-flavonoids include stilbene compounds, phenolic acids, and lignans; the glycosylation state of polyphenols and its diversity has a significant impact on the immunomodulatory activity, the sugar moiety, type, position, and extent of glycosylation in determining the anti-oxidant, anti-inflammatory, and the immunomodulatory activities of polyphenols. [79]. Polyphenols are prone to denaturation and oxidation due to the influence of their chemical structure and the external environment. Modern studies have confirmed that hydroxylation reduces flavonoid stability, while glycosylation, methylation, and acylation improve flavonoid stability. Moreover, the external environment, including pH, temperature, light, and oxygen, may change polyphenol properties [80]. For example, anthocyanins extracted from grapes appear red when pH < 7, blue when pH = 7, and blue or light blue when pH > 10 [81]. Therefore, the effects of the external environment on polyphenols during the extraction should be considered. Polyphenols have various biological activities, including anti-allergic, anti-oxidation, anti-bacterial, and anti-inflammation [82]. Natural polyphenols are abundant in grapes, blueberries, mangoes, and other fruits and vegetables, such as pearl vegetables, Chinese toon buds, coriander, and other vegetables [83]; these fruits and vegetables are the primary sources of polyphenols for humans.

### 2.3. Quinones

Quinnes share a cyclohexadienedione or cyclohexadiene dimethylene structure and are classified as quinones, naphthoquinones, and anthraquinones. Quinones are primarily derived from traditional Chinese medicines, such as the rhubarb of Polygonaceae, Polygonum multiflorum, Polygonum cuspidatum, Rubiaceae, Leguminosae cassia seeds, Senna, and Liliaceae aloe [84]. Emodin and alizarin are the most studied quinones in modern times. Emodin has an orange long needle like crystal with the chemical name 1-3-3-8-trihydroxy-6-methyl anthraquinone. Modern studies have confirmed that emodin has biological activities, such as hepato protection, anti-oxidation, and anti-bacterial [85], but it also has certain hepatotoxicity, nephrotoxicity, and reproductive toxicity. Emodin is a molecule that is fat soluble and water insoluble; thus, emodin-nicotinamide eutectic [86], emodin loaded nanoparticles [87], and emodin-PLGA film [88] can be developed to improve emodin bioavailability in vivo. Alizarin is an orange crystal or ochre yellow powder with the chemical name 1,2-dihydroxyanthraquinone. It has anticoagulants [58] and anti-cancer activities [89].

### 2.4. Pyrrole

Pyrrole compounds are abundant in plants and animals. The most widely studied natural pyrrole pigments include plant derived chlorophyll, phycobilin, animal-derived heme, and bilirubin. The common characteristic of the above four pigments is that they are all connected by four pyrrole rings. Chlorophyll can be divided into Chlorophyll a, Chlorophyll b, Chlorophyll c, Chlorophyll d, Chlorophyll f, prochlorophyll, and bacterial chlorophyll. It has a core porphyrin ring and a leaf alcohol chain, with a magnesium atom in the middle of the porphyrin ring [90]. Chlorophyll absorbs blue and red light from sunlight and reflects scatters of green light, making the plant’s leaves green. Chlorophyll is unstable in the external environment; a low pH, heat, light, oxygen, and enzyme reactions can also change the green properties. Therefore, the effects of the aforementioned conditions should be considered during the extraction and separation processes. Chlorophyll and its derivatives have anti-oxidation, anti-cancer, and anti-teratogenicity properties [91]. Phycobilin is a pigment in red algae and cyanobacteria that can be divided into phycocyanonbilin, phycoerythrobilin, and phycoviolobilin. Its common structure is linear tetrapyrrole, which differs from chlorophyll in that the leaf alcohol chain lacks a magnesium atom. Different phycobilins can exhibit red and blue colors due to differences in light absorption. The physical and chemical properties of phycobilin are easy to change under light conditions and an extreme pH. Phycobilin has anti-oxidant, anti-inflammatory, neuro-protective, and other biological activities [92].

### 2.5. Pyridines

Betaine is a water soluble pigment that contains a pyridine ring in *Caryophyllum*, including betaine and betaxanthin. Betaine is a reddish purple to dark purple: a pigment that causes plants to turn orange from yellow. Betaine has been widely used in food processing due to its good water solubility. More than 50 kinds of betaine and more than 30 kinds of betaxanthin have been identified [93]. Betaine is unstable at high temperatures, an extreme pH, with metal ions and light. Betaine has various biological activities, including anti-bacterial, anti-cancer, decrease blood lipid, liver protection, and neuroprotection [94].

## 3. Extraction Methods of Natural Plant Pigments

Traditional pigment extraction methods include solvent extraction, pressing, and impregnation. However, these methods have the problem of consuming many organic solvents, taking a long time, and having low yields. Therefore, some new pigment extraction methods have been developed to improve the above problems.

### 3.1. Supercritical Fluid Extraction

Supercritical fluid extraction is a technology that uses a supercritical fluid as an extractant to separate a component (extract) from its mixture (matrix). Most supercritical solvents are hydrocarbons, aromatics, alcohols, and some gases. CO_2_ is the most mainstream supercritical fluid because it is safe, lacks solvent residue after extraction, is cheap and easy to obtain, and can separate heat-sensitive compounds [95]. Co-solvent, pressure, and time effects on the extraction process should be considered in CO_2_ supercritical fluid extraction. Abrahamsson et al. [96] extracted carotenoids and chlorophyll A from microalgae at a temperature of 40~60 °C, a pressure of 15~30 MPa, with a liquid CO_2_ flow rate of 1–4 g/min, and co-solvent ethanol 0~0.2 mL/min. The extracted compounds were detected using a UV-vis spectrophotometer and modeled using the extractable amount and ethanol mole fraction. The carotenoid yield was 0.25 mg/g, the chlorophyll A yield was 0.96 mg/g, and the extraction amount depended on the co-solvent amount. DaPorto et al. [97] extracted polyphenols from white grape seeds at 40 °C, different pressures, CO_2_ fringes, and co-solvent ratios. The optimal extraction conditions were as follows: the highest total polyphenol concentration was obtained at 80 bar pressure, 6 kg/h of the CO_2_ flow rate, and 20% (*w*/*w*) of the co-solvent. Combined with previous research, increasing pressure can increase the yield, but not significantly.

### 3.2. Ultrasound-Assisted Extraction

The purpose of ultrasonic-assisted extraction technology is to make the effective components of the extracted material quickly enter into the solvent under the action of an ultrasonic wave to obtain the multi-component mixed extract and then separate, refine, and purify the extract using appropriate methods. This is a new technology to obtain the required monomer chemical components. Ultrasonic waves can produce comprehensive effects, such as cavitation, vibration, crushing, and mixing, in the medium. Ultrasound can break the cell wall and accelerate the extract dissolution. Ultrasound-assisted extraction has often been used to extract thermally unstable active substances. The primary advantages are shortening the extraction time, improving extraction efficiency, a wide application, and simple operation [98]. Sahin et al. [99] used the ultrasound-assisted extraction of lutein and carotenoids from Chlorella, added them to chitosan to prepare chitosan films, and characterized them using Fourier transform infrared spectroscopy and SEM technology. The results display how 4.844 ± 0.78 mg/g lutein and 0.533 ± 0.06 mg/g carotenoids can be prepared using ultrasonic extraction. The pigment–chitosan complex has anti-antioxidant activity and can be used as a wound-healing plaster. Zhong et al. [100] used the ultrasound-assisted extraction of diosgenin; the results presented that when the acetone volume fraction was 74%, the extraction time was 31 min and the temperature was 54 °C, while the dioscorea pigment yield was the highest (32.27%). The extracted dioscorea pigment exhibited good anti-oxidant activity.

### 3.3. Microwave-Assisted Extraction

Microwave-assisted extraction mainly depends on the heating effect of microwaves to obtain a higher temperature and mass transfer rate. The greater the solvent polarity, the faster the microwave energy absorption and heating up, accelerating the extraction rate. The primary advantages are speed and efficiency, uniform heating, solvent saving, and selectivity. The solvent used for the extraction, microwave time, stirring, and temperature influenced the microwave extraction. Long term microwave heating can change the extract’s properties [101]. Sharma et al. [102] used the microwave-assisted extraction method to extract betaine and betaaxanthin from tricolor amaranth leaves and characterized them using Fourier transform infrared spectroscopy. The highest betacyanin yield was 71.95 mg/g when the microwave power was 450 W, and the temperature was 90 °C for 15 min, while the beet flavin yield was the highest (42.30 mg/g) when the microwave power was 200 W, and the temperature was 60 °C. Fernandez-Aulis et al. compared the three methods of impregnation, ultrasonic extraction, and microwave-assisted extraction from corn. The results exhibited that the anthocyanin yield in the corn husk was 21.89 ± 1.23 mg/g after 30 min, 25.80 ± 0.59 mg/g using the ultrasonic method after 20 min, and 24.47 ± 0.85 mg/g using the microwave-assisted method after 1 min. The microwave-assisted method greatly shortened the extraction time [103].

### 3.4. Enzyme-Assisted Extraction

The enzyme hydrolyzes the plant cell wall and destroys the cell matrix as a biological reaction catalyst, promoting the extraction material into the solvent, accelerating the extraction rate, and increasing the yield. The advantages of enzyme-assisted extraction included a short extraction time, strong specificity, mild reaction conditions, and high yield. Enzyme selection was the most important aspect of enzyme-assisted extraction. Enzymes, such as cellulase, hemicellulase, ligninase, pectinase, and amylase, could be utilized for enzyme-assisted extraction. One enzyme could be used for extraction, or a mixture of multiple enzymes could be used for the extraction [104]. Zhao et al. [105] investigated the optimal conditions for extracting astaxanthin from Haematococcus pluvialis using cellulase and Pectinase. The astaxanthin yield was 67.15% at 45 °C, pH 5.0, and 1.0% cellulase for 6 h. When the content of Pectinase was 0.08%, the astaxanthin yield was 75.30% at 55 °C, pH 4.5. Lombardelli et al. [106] extracted betaine from beetroots using an enzyme blend of cellulase, xylanase, and pectinase. After optimizing the conditions, such as the amount of mixed enzyme, the treatment time, and temperature, the optimum conditions for extracting betaine with mixed enzyme were determined: an enzyme amount of 25 U/g, a temperature of 25 °C, and a treatment time of 240 min.

### 3.5. Ultra High Pressure Assisted Extraction

The ultrahigh pressure extraction technology uses high pressure to penetrate the solvent into the raw material, allowing the target compound in the cell to dissolve in the solvent. After a period under high pressure, the target compound reached a dissolution equilibrium in the solvent. Afterward, the pressure returned to normal, and the target compound spread to the extract around the tissue. Simultaneously, ultrahigh pressure destroyed the tissues, promoting target compound release [107]. Nunes et al. [108] studied the extraction of the beet pigment from cactus by high pressure CO_2_-acidified water extraction using the response surface optimization method. The results indicate a maximum yield of 89 ± 0.7 mg/100 g of beet pigment at 100 bar, 40 °C, and a 20% solid–liquid mixture/pressurized CO_2_. Ferrari et al. [109] compared anthocyanin and flavonoid extraction from new pigment rice Nerone Gold 26/6 using high pressure extraction, ultrasound-assisted extraction, and conventional solid–liquid extraction. High pressure extraction yielded the highest yield of the total flavonoids, while ultrasound assisted extraction yielded the highest yield of the anthocyanins, and both methods produced higher yields than conventional solid–liquid extraction.

### 3.6. Joint Application

Although the above extraction methods have advantages, they also have limitations, such as high cost and low equipment utilization, therefore, several studies used the above extraction methods in series to overcome these limitations. Shahram et al. [110] combined ultrasound-assisted and enzyme-assisted extraction and used the response surface optimization method to extract β-carotene from a citrus processing residue. The study determined that the β-carotene yield and the anti-antioxidant activity were the highest at a Pectinase concentration of 0.4%, an ultrasound at 115.5 min, and a pH of 5.11. Fu et al. [111] established a microwave-enzyme-assisted aqueous two phase extraction method to extract the total polyphenols and lutein from marigolds. The total polyphenols yield was 84.61 mg/g, the corresponding recovery rate was 95.35, the lutein yield was 7.32 mg/g, and the corresponding recovery rate was 99.85%. The yield was significantly increased compared to Soxhlet extraction, microwave-assisted extraction, and enzyme-assisted extraction. Therefore, pigment extraction cannot be limited to a single technique, and various techniques can be utilized.

## 4. Biological Activity of Natural Plant Pigments

### 4.1. Anti-Oxidation

Reactive oxygen species and reactive nitrogen play an important role in signal transduction and the maintenance of balance in human cells at medium/low concentrations. However, they also lead to oxidative stress at high concentrations, resulting in damage to the cellular structure, including lipids, membranes, proteins, DNA, cardiovascular diseases, cancer, nervous system disorders, diabetes, and stroke [112]. Modern studies have confirmed that natural plant pigments can scavenge reactive oxygen species and increase superoxide dismutase activity through Keap1/Nrf2, AMPK/Nrf2, and other signaling pathways to exert anti-antioxidant effects. Josson Akkara et al. [113] used β-carotene to treat liver injury induced by bromobenzene and compared its therapeutic effect with the standard hepatoprotective drug silymarin. The results indicated that β-carotene could reduce oxidative stress and bromobenzene induced liver injury by reducing peroxide and inflammatory cytokine levels while increasing oxidant levels. This therapeutic effect is similar to that of silymarin. Wang et al. [114] used anthocyanin in a carbon tetrachloride-induced liver injury model in mice to study the anti-antioxidant effect of anthocyanin and its mechanism. The results exhibited how anthocyanins could reduce liver injury in mice by reducing malondialdehyde (MDA), interleukin-6 (IL-6), and interleukin-1 β (IL-1 β). It may be that in this mechanism, anthocyanins exert anti-antioxidant and anti-inflammatory effects via the Keap1/Nrf2 pathway. Yu et al. [115] confirmed that cyanidin-3-glucoside could scavenge reactive oxygen species via the AMPK/Nrf2 pathway, enhance the anti-oxidation defense ability of mice, weaken H_2_O_2_-induced apoptosis, and prevent liver injury. Brotosudarmo pointed out in their study that the hydroxyl and ketone groups in the astaxanthin structure can play a role in neutralizing reactive oxygen species, quenching harmful singlet oxygen, reducing the formation of free radicals, and thus exerting anti-antioxidant effects [116].

### 4.2. Anti-Inflammation

Inflammation is caused by tissue damage or pathogen activation by the innate immune system after the injury or infection of the human body, inducing leukocyte synthesis and removing inflamed sites and tissue regeneration. Inflammation is usually temporary; however, when it becomes chronic, it can lead to numerous diseases. Modern studies have confirmed that inflammation is associated with many diseases, such as asthma, cancer, and atherosclerosis [117]. Natural plant pigments can delay the inflammation process by reducing the expression of inflammatory factors such as tumor necrosis factor-α(TNF-α) and interleukin. Takahashi et al. [118] investigated the anti-inflammatory effect of β-carotene in atopic dermatitis induced by a low zinc/magnesium diet. Studies have confirmed that β-carotene can reduce skin inflammation by inhibiting inflammatory factors such as IL-1 β, reducing matrix metalloproteinases activity, and promoting fibroin expression. Blas-Valdivia et al. [119] studied the cardio-protection effect of C-phycocyanin and its mechanism using isoproterenol induced acute myocardial infarction in rats. The results displayed that C-phycocyanin could reduce cytokines in the contents, such as TNF-α, reactive oxygen species, IL-β, interferon-γ, myocardial enzymes, and creatine kinase, and play the role of anti-inflammation and anti-oxidation. Li et al. [120] studied the benefits of curcumin on traumatic brain injury and its mechanism. The results revealed that curcumin has an anti-inflammatory effect, inhibiting IL-1 β, IL-6, TNF-α, and p38 expressions, thus alleviating traumatic brain injury.

### 4.3. Anti-Cancer

Current cancer treatments include chemotherapy and other induced irreversible metabolic damage to kill cancer cells; however, chemotherapy still has serious side effects. Therefore, it is necessary to identify new ways to treat cancer. Modern studies have confirmed that natural plant pigments can induce the apoptosis of cancer cells through signaling pathways such as P13K/AKT and ERK1/2MAPK, thereby exerting anti-cancer effects [121]. Cui et al. [122] investigated the anti-cancer effect of astaxanthin and established an N-nitroso-benzylamine induced rat esophageal cancer model. The results indicated that astaxanthin cereal significantly reduced the incidence of esophageal cancer by increasing glutathione peroxidase and superoxide dismutase activities and reducing NF-κB and COX2 protein expressions. Yeh et al. [123] confirmed that naphthoquinone shikonin extracted from shikonin could induce apoptosis and reduce human alveolar basal epithelial cells A549 proliferation in a dose dependent manner via a p53 mediated signal pathway. Lim et al. [124] studied the preventive effect of delphinin in anthocyanins on epithelial ovarian cancer. The results demonstrated that vermicellin induced the apoptosis of ovarian cancer cells by fragmenting DNA at the SKOV3 point and inhibited SKOV3 cell proliferation via two signaling pathways: P13K/AKT and ERK1/2MAPK line.

### 4.4. Neuro Protection

The incidence of nervous system diseases is relatively high. Stroke, epilepsy, senile dementia, and other common nervous system diseases affect the health of tens of millions of people worldwide. Coupled with rare diseases, such as Wright syndrome, and Rasmussen encephalitis, it has become an increasingly severe medical burden and world problem [125]. Studies have confirmed that curcumin could inhibit the Wnt signaling pathway to inhibit apoptosis and the inflammation of neurons when exposed to hypoxia/oxygen reperfusion and play a neuroprotective role [126]. Jiang et al. [127] revealed that curcumin can inhibit nitric oxide synthase and nitrite/nitrate production, prevent the injury of cerebral capillary endothelial cells, reduce the infarct volume, and improve neurological function, confirming the protective effect of curcumin on cerebral ischemia/reperfusion injury. Gazzin et al. [128] examined the neuro-protection effect of curcumin in the spontaneous model of neonatal hyperbilirubinemia. The results illustrated that curcumin mediated multiple targets of neuro injury (inflammation, redox imbalance, and glutamate neurotoxicity), allowing the cerebellum to develop completely and recover abnormal rat behavior. Wang et al. [129] confirmed that shikonin could decrease the neurological deficit score, reduce the infarct size and MDA content, and increase superoxide dismutase and catalase contents in shikonin-treated mice, thus playing a neuroprotective role. Phycocyanin and its tetrapyrrole chromophore phycocyanin from spirulina improved multiple sclerosis by improving neuroinflammation and preventing demyelination and axonal loss [130]. Phycocyanin also has great potential in treating Alzheimer’s disease [131].

### 4.5. Cardiovascular Protection

Cardiovascular disease is a circulatory system disease that affects the heart, arteries, and veins and is commonly related to arteriosclerosis. Coronary heart disease, hypertension, angina pectoris, and myocardial infarction are the major diseases. Recently, many natural products have been developed to treat vascular diseases [132]. Zhou et al. [133] studied the mechanism of the cardiovascular protective effect of curcumin. The results exhibited that curcumin could down regulate lncRNAH19 expression and inhibit Wnt/β-catenin expression, thus inhibiting vascular restenosis induced by intimal hyperplasia and protecting the cardiovascular system. Qian et al. [134] demonstrated that anthocyanins could alleviate cisplatin induced heart injury by inhibiting apoptosis when mediated by reactive oxygen species and the extracellular regulated kinase signal pathway. Thrombosis is partly attributed to the activation of endothelial tissue factors, leading to vascular thrombosis. Lee and others [135] have confirmed that carotenoids can inhibit the activity of tissue factors in endothelial cells by promoting protein kinase B (Akt) phosphorylation. Ferreira Santos et al. [136] studied the therapeutic effect of lycopene on angiotensin II induced hypertensive rats. The results revealed that lycopene could improve hypertension and cardiovascular remodeling caused by angiotensin II; however, it did not affect blood pressure in normal rats, confirming the therapeutic potential of lycopene in hypertension.

## 5. Modification of Natural Plant Pigments

Natural phytochromes have limited applications due to their instability. Lipid carriers, protein nanoparticles, chitosan nanoparticles, and metal ions have been used to modify and stabilize natural phytochromes; these methods can improve the stability of natural plant pigments, thereby increasing their application range. Table 2 lists the materials used in various methods and their advantages.

### 5.1. Lipid Nanoparticles

Lipid-based colloidal carriers can improve the fat soluble natural compounds’ solubility in water, bioavailability in vivo, and easily degradable compound stability. Therefore, they are widely used in the carrier research of natural compounds. Lipid nanoparticles can be divided into solid lipid nanoparticles with the solid lipid as the lipid core and nanostructured lipid carriers with a liquid lipid as the lipid core [141]. Osanlou et al. [142] prepared zeaxanthin solid lipid nanoparticles and nanostructured lipid carriers by combining high shear homogenization and an ultrasound. According to studies, zeaxanthin loaded lipid nanoparticles are spherical; zeaxanthin is wrapped in the lipid material but has no chemical interaction with the material. Tamjidi et al. [143] displayed that the nanostructured lipid carriers loaded with astaxanthin had good stability in simulated gastric juice and could increase astaxanthin’s bioavailability. Pelissari et al. [144] used spray cooling technology to prepare solid lipid particles loaded with lycopene/sunflower oil and shortening as a carrier. Studies have revealed that combining Arabic gum and the carrier could reduce lycopene degradation in low temperature and vacuum environments [145]; prepared nanostructured lipid carriers loaded with curcumin and studied the anti-cancer effect of the nanosystems. The results indicate that these nanoparticles could achieve the sustained release of curcumin and have a more sustained anti-cancer effect than free curcumin.

### 5.2. Protein Nanoparticles

Proteins have been widely used to study drug carriers due to their good biocompatibility and degradability. The proteins used in the delivery system include albumin, whey protein, and bean globulin. These proteins have a good loading capacity and sustained release [146]. Liu et al. [147] exposed that sodium caseinate, whey protein isolate, and soybean protein isolate could successfully load annatto and increase its stability and water solubility. In simulated cheese production, soybean protein isolate nanoparticles loaded with annatto could inhibit pigment penetration into whey, providing a theoretical basis for their application in food. Radomirovic et al. [148] have studied phycocyanin-β lactoglobulin nanoparticle stability. Studies have revealed that β-lactoglobulin loaded with phycocyanin can reduce the trend of heat induced polymerization and the fibril formation of proteins, presenting higher stability. Gulsu et al. [149] demonstrated that the entrapment efficiency of bovine serum albumin particles, when loaded with the anti-cancer drug doxorubicin, was 75%, and the sustained release effect lasted for up to 96 h. The particles could reduce the cancer cell activity to a greater extent than free doxorubicin; thus, the nanoparticles were capable of delivering doxorubicin.

### 5.3. Chitosan Nanoparticles

Chitosan is a natural polymer with easy structure modification, low cytotoxicity, good biocompatibility, and biodegradability; therefore, it is a good choice as a carrier [150]. Chitosan can be used as a pigment carrier in pigment extraction due to its good adsorption. Tanabtabzadeh et al. [151] confirmed that chitosan adsorption could extract betaine from beetroot plants. Xue et al. [152] prepared chitosan microspheres loaded with R-phycoerythrin using the suspension crosslinking method and studied the effect of adding Agar to the microspheres on R-phycoerythrin release. The results displayed that R-phycocyanin has a maximum loading rate at pH 3.59, and this loading rate increases with an increase in the temperature and solution salt ion concentration. Chitosan can achieve the sustained release of R-phycoerythrin, and the release rate can be accelerated after adding agarose; therefore, the release rate of R-phycoerythrin can be adjusted according to the amount of agarose in the particles. Gandia-Herrero et al. [153] used spray drying procedures to encapsulate betaine into chitosan, which protected the anti-antioxidant activity of betaine for six months. The above studies confirmed the potential of chitosan for extraction, encapsulation, and preservation of pigments.

### 5.4. Metal Ions

Dietary or intestinal microorganisms produce metal nanoparticles in human or animal blood and tissues. These are primarily single atoms of metals, such as gold, silver, iron, and platinum, or protein nanoparticles combined with proteins. These endogenous metal nanoparticles have been found to inhibit cancer cell growth and reproduction. Therefore, metal nanoparticles as transport carriers have unique physical and chemical properties [154]. Venil et al. [155] treated keraxanthin extracted from Sanguisorba officinalis with a silver nitrate solution to prepare silver nanoparticles loaded with cornaxanthin. The nanoparticles demonstrated significant cytotoxicity to the human keratinocyte line with potential wound-healing properties. Sigurdson et al. [156] confirmed that the stability of acylated anthocyanins was significantly improved after forming chelates with aluminum and iron ions. Davaeifar et al. [157] synthesized phycocyanin–zinc oxide nanorods. The phycocyanin-coated zinc oxide nanorods had good thermal stability and less cytotoxicity to fibroblasts L929. In summary, metal nanoparticles have great potential for improving pigment stability and as carriers for pigment transport.

## 6. Prospect

Natural pigments are widely used in cosmetics and food industries due to their wide variety, different colors, and high safety; for example, Pepha^®^-Ctive containing β-carotene was launched by Pentapharm Ltd., Linablue^®^ containing phycocyanin was launched by Dainippon Ink and Chemicals Inc., AstaPure^®^ containing astaxanthin was launched by Algatech and FucoVital TM containing fucoxanthin [158].

However, natural plant pigments have problems, such as poor stability and solubility. Additionally, the practical application of natural pigments has numerous problems. First, the number of natural colorants approved by authoritative national organizations for food and beverages is limited. Second, there are no clear standards for using some pigments. The scope of application and dosage without detection standards could lead to the abuse and overuse of pigments, resulting in harmful events. Finally, a natural pigment yield was low due to the limitations of extraction technology, resulting in a large waste of resources, and its practical application was limited. Modern pharmacological studies have confirmed that natural pigments have various biological activities and can be used for treating different diseases. However, their application is now limited to the food and cosmetic industries, and few of them have been used in the pharmaceutical industry. Therefore, its pharmacological effect cannot be fully realized. This study reviewed the chemical classification, extraction methods, biological activities, and modification methods of natural plant pigments to expand the natural plant pigment application in medicine, food, and cosmetics. These different extraction technologies increased the natural pigment yield while preventing degradation and oxidation during extraction. These technologies reduced the use of organic solvents while increasing the environmental protection and safety of the extraction process. Additionally, more mature studies confirmed that pigment stability could be improved by wrapping pigments into different nanoparticles in order to prolong the service life of natural pigments, which helps to increase the application of the natural pigment in different ways.

The rich and diverse plant resources in nature provide a large number of natural pigments. In recent years, people’s demand for natural plant pigments has increased. With the development of production technology, the largescale preparation of natural plant pigments tends to mature. The commercial application of products based on natural plant pigments is becoming more promising with potential economic benefits.

## Figures and Tables

**Table 1:** Chemical classification, chemical structure, and biological activity of natural plant pigments.

Classification	Natural Plant Pigment		Chemical Constitution	Bioactivity	Reference
Carotenoids	β-carotene		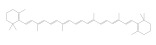	Anti-inflammation	[9]
				Anti-oxidation	[10]
				Anti-cancer	[11]
	Lycopene		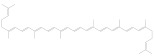	Anti-oxidation	[12]
				Anti-inflammation	[13]
				Anti-tumor	[14]
				Anti-atherosclerosis	[15]
	Astaxanthin		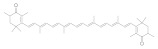	Anti-inflammation	[16]
				Neuro protection	[17]
				Anti-fibrosis	[18]
				Anti-cancer	[19]
				Anti-oxidation	[20]
	Lutein		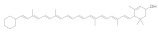	Cardiovascular protection	[21]
				Anti-oxidation	[22]
				Neuro protection	[23]
				Anti-cancer	[24]
	Zeaxanthin		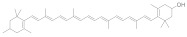	Anti-inflammation	[25]
				Anti-oxidation	[26]
				Anti-cancer	[27]
				Neuro protection	[28]
Polyphenols	Anthocyanins	Pelargonidin	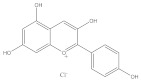	Anti-obesity	[29]
				Anti-cancer	[30]
				Anti-oxidation	[31]
				Anti-inflammation	[32]
		Cyanidin	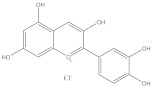	Anti-oxidation	[33]
				Bone protection	[34]
				Anti-inflammation	[35]
				Anti-cancer	[36]
		Delphindin	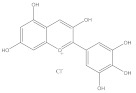	Anti-cancer	[37]
				Anti-inflammation	[38]
				Anti-oxidation	[39]
				Neuro protection	[40]
		Peonidin	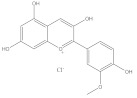	Bone protection	[41]
				Anti-inflammation	[42]
		Petunidin	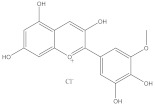	Anti-inflammation	[43]
				Bone protection	[44]
				Cardiovascular protection	[45]
		Malvidin	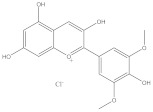	Anti-cancer	[46]
				Anti-inflammation	[47]
				Cardiovascular protection	[48]
				Anti-oxidation	[49]
	Curcumin		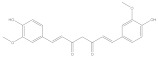	Anti-inflammation	[50]
				Anti-cancer	[51]
				Neuro protection	[52]
				Anti-bacterial	[53]
Quinone	Emodin		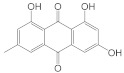	Anti-cancer	[54]
				Anti-fibrosis	[55]
				Anti-inflammation	[56]
				Neuro protection	[57]
	Alizarin		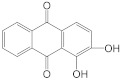	Anti-coagulation	[58]
				Anti-bacterial	[59]
				Anti-cancer	[60]
Pyrrole	Chlorophyll A		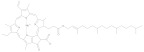	Antigen toxicity	[61]
				Anti-inflammation	[62]
				Anti-oxidation	[63]
	Chlorophyll B		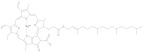	Antigen toxicity	[61]
				Anti-oxidation	[63]
	Phycocyanobilin		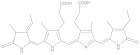	Anti-inflammation	[64]
				Anti-oxidation	[65]
				Neuro protection	[66]
	Phycoerythrobilin		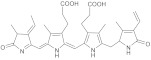	Anti-oxidation	[67]
Pyridines	Betacyanin		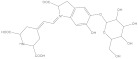	Anti-hypersensitivity	[68]
				Anti-inflammation	[69]
				Anti-thrombotic	[70]
				Anti-oxidation	[71]
	Betaxanthin		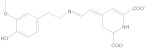	Anti-tumor	[72]
				Anti-oxidation	[73]

**Table 2:** Modification methods of natural plant pigments.

Modification Method	Type/Material	Merit	Reference
Lipid carrier	Solid lipid nanoparticles	High biocompatibility; enhanced membrane permeability; enhanced solubility; biodegradability; increased intestinal drug dilution	[137]
	Nanostructured lipid carriers		
Protein nanoparticle	Soy isolate protein	Rich sources, biocompatibility, biodegradability; the synthesis process is simple	[138]
	Albumin		
Chitosan nanoparticles	Deacetylated chitin	Biodegradability, biocompatibility, bioactivity, non-toxicity, polycation	[139]
Metal	Au	High biocompatibility, high biodegradability, high anti-bacterial activity, targeted delivery, controlled release	[140]
	Ag		
	ZnO		
	Cu and oxides		

## Data Availability

Data sharing is not applicable to this article.
